# Modulation of endoplasmic reticulum stress via sulforaphane-mediated AMPK upregulation against nonalcoholic fatty liver disease in rats

**DOI:** 10.1007/s12192-022-01286-w

**Published:** 2022-07-02

**Authors:** Somaya Z. Mansour, Enas M. Moustafa, Fatma S. M. Moawed

**Affiliations:** 1 Radiation Biology Research, National Center for Radiation Research and Technology, Atomic Energy Authority, Cairo, Egypt; 2 Health Radiation Research, National Center for Radiation Research and Technology, Egyptian Atomic Energy Authority, Cairo, Egypt

**Keywords:** NAFLD, Ionizing radiation, Endoplasmic reticulum stress, Sulforaphane, AMPK, PPAR-α

## Abstract

Nonalcoholic fatty liver disease (NAFLD) is a major health concern. Endoplasmic reticulum (ER) stress, inflammation, and metabolic dysfunctions may be targeted to prevent the progress of nonalcoholic fatty liver disease. Sulforaphane (SFN), a sulfur-containing compound that is abundant in broccoli florets, seeds, and sprouts, has been reported to have beneficial effects on attenuating metabolic diseases. In light of this, the present study was designed to elucidate the mechanisms by which SFN ameliorated ER stress, inflammation, lipid metabolism, and insulin resistance — induced by a high-fat diet and ionizing radiation (IR) in rats. In our study, the rats were randomly divided into five groups: control, HFD, HFD + SFN, HFD + IR, and HFD + IR + SFN groups. After the last administration of SFN, liver and blood samples were taken. As a result, the lipid profile, liver enzymes, glucose, insulin, IL-1β, adipokines (leptin and resistin), and PI3K/AKT protein levels, as well as the mRNA gene expression of ER stress markers (IRE-1, sXBP-1, PERK, ATF4, and CHOP), fatty acid synthase (FAS), peroxisome proliferator–activated receptor-α (PPAR-α). Interestingly, SFN treatment modulated the levels of proinflammatory cytokine including IL-1β, metabolic indices (lipid profile, glucose, insulin, and adipokines), and ER stress markers in HFD and HFD + IR groups. SFN also increases the expression of PPAR-α and AMPK genes in the livers of HFD and HFD + IR groups. Meanwhile, the gene expression of FAS and CHOP was significantly attenuated in the SFN-treated groups. Our results clearly show that SFN inhibits liver toxicity induced by HFD and IR by ameliorating the ER stress events in the liver tissue through the upregulation of AMPK and PPAR-α accompanied by downregulation of FAS and CHOP gene expression.

## Introduction

Nonalcoholic fatty liver disease is defined as a fatty change (steatosis) affecting greater than 5% of hepatocytes, enlargement of the liver (hepatomegaly), and inflammation (steatohepatitis) (Cobbina and Akhlaghi 2017). The prevalence of NAFLD is increasing all over the world, especially in Western countries (Yu et al. 2019). Therefore, NAFLD has the potential to be the most common cause of chronic liver disease in the near future. NAFLD does not have any clinical drug therapy. A variety of stressors have the potential to destroy organs or signal distribution routes, leading to genetic anomalies, functional impairment, and/or diseases. According to recent research, ionizing radiation and being overweight bring serious health risks (Moustafa et al. 2021). Meanwhile, the liver is easily influenced by many environmental conditions, such as ionizing radiation. Living organisms exposed to relatively high-dose radiation can sustain severe damage or die within a short period due to acute effects (Chiba et al. 2002). Once gamma radiation strikes the body, it induces ionized particles and excitation in the tissues, disrupting cellular functions. As a consequence, physiological destruction to the body will take place, with the lethality of this injury defined by a variety of factors such as the type and energy of the radiation, the overall dosages and dose rate, the impact and body part subjected, the aging process exposed to radiation, and the radiation responsivity of the organ exposed (Reisz et al. 2014). The effects of radiation on the liver can be impacted by lifestyle factors such as obesity, nutrition, and alcohol consumption, all of which are linked to a variety of liver diseases (Akiba and Mizuno 2012). Mechanisms underlying the basis of radiation impacts on the liver will lead to a variety of applications that will make therapy more effective. Appropriate agents promote anti-inflammatory or anti-oxidative properties in the liver, potentially lowering metabolic disorders as well as the negative consequences of radiation exposure (Nakajima et al. 2018).

The molecular mechanisms underlying fatty liver are not fully understood. Dysregulation of hepatic lipid homeostasis caused by pathological conditions such as reduced fatty acid oxidation, enhanced de novo lipogenesis, elevated hepatic fatty acid influx, and/or increased systemic insulin resistance is thought to be important in the development of the fatty liver. Indeed, therapies aimed at reducing body weight and/or alleviating insulin resistance reduce the fatty liver. AMP-activated protein kinase (AMPK) is an intracellular fuel sensor important in the regulation of lipid metabolism. In the liver, activation of AMPK leads to increased fatty acid oxidation and simultaneously to decreased lipid synthesis. Of interest, antidiabetic drugs, including metformin and the thiazolidinediones, alleviate fatty liver in humans and rodents by regulating lipid metabolism through AMPK activation. Thus, AMPK represents an attractive target for therapeutic intervention in the treatment of hepatic disorders (Shen et al. 2013).

Evidence suggests that ER stress plays an important role in the development of NAFLD. As a cytoplasmic organelle, the endoplasmic reticulum (ER) is responsible for folding, synthesizing, and modifying proteins. If ER cannot carry out its functions, unfolded or misfolded proteins accumulate in the cell. This pathological situation is called ER stress. This stress triggers the response known as the “unfolded protein response” (UPR) in the cell. With this response regulated by chaperones, the proper folding capacity of proteins is enhanced and the accumulation of misfolded proteins is prevented to some extent. UPR is carried out through major chaperones called glucose-regulating protein 78 kDa (GRP78) and three ER transmembrane receptors, including activating-transcription factor 6α (ATF6α), inositol-requiring enzyme 1 α (IRE1α), and protein kinase-R-like ER kinase (PERK). Each of these proteins activates specific pathways that enhance the accurate folding capacity of proteins and accelerate the degradation of misfolded proteins (Xu et al. 2005).

Endoplasmic reticulum stress-induced IRE1α increases the synthesis of X-box-binding protein-1 (XBP-1), which is associated with many regulatory pathways. Evidence suggests that ER stress plays an important role in the development of NAFLD (Li et al. 2012; Pagliassotti 2012). The modulation of ER stress is important in the treatment of this disease. The modulation of ER stress is important in the treatment of this disease. Sulforaphane (SFN) derived from the hydrolysis of glucoraphanin has been reported to have important medicinal value. SFN has been reported to exhibit antioxidant, neuroprotective, and anticancer properties (Guerrero-Beltran et al. 2012; Liang and Yuan 2012). It has also been reported that SFN has the potential to fight obesity by activating the AMPK signaling pathway (Lee et al. 2012; Choi et al. 2014; Yao et al. 2015). Although several studies have investigated the anti-obesity properties exerted by SFN, the molecular mechanism underlying the protective role of this compound on lipotoxicity and glucotoxicity in NAFLD remains unknown. In the present study, we exploited a rat model of NAFLD to evaluate the effect of SFN on the high-fat diet (HFD) and/or ionizing-irradiation-induced hepatic oxidative damage. The study also aimed to determine the mechanisms responsible for the therapeutic effect of SFN by investigating the expression of genes related to ER stress, lipid metabolism, and insulin resistance.

## Materials and methods

### Materials

Sulforaphane was obtained from Source Naturals (Scotts Valley, CA, USA). All other chemicals were purchased from Sigma-Aldrich (St. Louis, MO, USA).

### Irradiation process

Whole-body ionizing irradiation (IR) was performed at the National Centre for Radiation Research and Technology (NCRRT, Cairo, Egypt) using Canadian gamma cell-40 (^137^Cesium) at a dose rate of 0.67 Gy min^−1^ for a total dose of 6 Gy (Ramadan et al. 2001).

### Animals

Thirty male albino Wistar rats were provided from the breeding unit of the Egyptian Holding Company for Biological Products and Vaccines provided (Giza, Egypt). The rats were 5 weeks old, weighed 130 to 150 g, and were kept in conventional cages (six animals per cage). The animals were kept in an air-conditioned (25 ± 2 °C) environment with unrestricted access to water and a regular laboratory feed (El-Nasr Co. Cairo, Egypt). They were also treated to a 12:12-h light–dark cycle. All the experimental procedures were carried out according to the principles and guidelines of the Ethics Committee of the National Research Centre conformed to the “Guide for the care and use of Laboratory Animals” for the use and welfare of experimental animals, published by the US National Institutes of Health (NIH publication No. 85–23, 1996).

### Experimental design

After a 1-week acclimation period, some of the animals were fed normal chow, while others were fed HFD for 8 weeks. HFD was provided by El-Nasr Co. (Cairo, Egypt), which comprised 50% carbohydrates/starch, 27% fat, 10% protein, 10% sucrose, 1.5% fiber, and 1.5% vitamins. Rats were allocated and randomly divided into five equal groups with 6 rats each to enable differences in treatment to be determined with statistical significance (*p* < 0.05) as determined using the G-power statistical program.

The animal groups were as follows:

- *Group 1* (control): rats receiving standard chow.
- *Group 2* (HFD): rats were fed with HFD.
- *Group 3* (HFD + SFN): HFD rats were treated with SFN orally by gavage at daily doses of 10 mg/kg B. W dissolved in distilled water (Tian et al. 2021) for 4 weeks.
- *Group 4* (HFD + IR): rats were fed with HFD, and exposed to fractionated doses of IR (3 × 2 Gy) up to a total exposure of 6 Gy (Kumar et al. 2017) to exasperate metabolic syndrome.
- *Group 5* (HFD + IR + SFN): HFD rats were exposed to IR as in group 4, and treated with SFN as in group 3.

Throughout the experiment, the body weight of the rats was recorded, before and after the administration of drugs. Blood samples were collected via retro-orbital bleeding into tubes. Fasting blood glucose was estimated then at − 80 °C and the blood aliquots were stored for further analysis. Then rats were sacrificed by cervical dislocation and liver samples were collected and divided into two portions; the first one was stored at − 80 °C for assessment of oxidant/antioxidant biomarkers, pro-inflammatory mediators, and gene expression. Other portions of liver samples were fixed in 10% neutral formalin and prepared for histopathological examination.

### Biochemical analyses

Using kits supplied by BIOMED, the activities of serum alkaline phosphatase (ALP), alanine aminotransferase (ALT), and aspartate amino transaminase (AST) were assessed. The free fatty acid assay was performed in serum with BioVision’s Free Fatty Acid Quantification Colorimetric Kit. A commercial kit (BIOMED, Cairo, Egypt) was used to measure serum glucose levels. Total cholesterol (TC), total triglycerides (TG), low-density lipoproteins (LDL-c), and high-density lipoprotein (HDL-c) in serum were measured using commercial kits from RANDOX Reagents (USA). Lipid peroxidative products were measured using the thiobarbituric acid test for malondialdehyde (MDA) in liver tissue, as described by Satoh (1978). Superoxide dismutase (SOD) activity was determined in liver tissue spectrophotometrically (Marklund 1992). Reduced glutathione (GSH) contents were measured in liver tissue according to the method of Ahmed et al. (1991), while the activity of the catalase enzyme was determined in liver tissue according to Aebi (1984). Serum IL-1β concentration was assayed by using ELISA kits for rats (Glory Science Co., Ltd., Del Rio, TX, USA). Rat Phosphotylinosital 3 Kinase (PI3K) and rat phospho-AKT (Ser473) in liver tissue were estimated using MyBioSource (San Diego, CA 92,195-3308USA). Also, insulin serum levels were evaluated (Merck Millipore, Billerica, MA, USA) using an enzyme-linked immunosorbent assay (ELISA)**.** Serum resistin and leptin were measured using an ELISA kit (R&D Systems). To estimate the homeostasis model assessment for insulin resistance (HOMA-IR), the following equation was used, fasting insulin × fasting glucose/405 (Roza et al. 2016).

### Detection of gene expression by real-time quantitative polymerase chain reaction (PCR)

#### Isolation of RNA and reverse transcription

The mRNA expression of activating transcription factor 4 (ATF4), inositol-requiring enzyme-1 (IRE-1α), adenosine monophosphate-activated protein kinase (AMPK), spliced X-box binding protein 1 (sXBP1), protein kinase R (PKR)-like endoplasmic reticulum kinase (PERK), peroxisome proliferator-activated receptor-alpha (PPAR-α), fatty acid synthase (FAS), and C/EBP–homologous protein (CHOP) were examined. Using the TRIzol reagent (Life Technologies, USA) according to the manufacturer’s instructions, total RNA was isolated from 30 mg of liver tissues. Agarose gel electrophoresis (1%) was used with ethidium bromide staining to confirm the integrity of RNA. Synthesis of the first-strand complementary DNA (cDNA) was achieved with reverse transcriptase (Invitrogen) using 1 μg total RNA as the template, according to the manufacturer’s protocol. RT-PCRs were performed using the Sequence Detection Program (PE Biosystems, CA) in a thermal cycler stage one plus (Applied Biosystems, USA). A 25-μL total volume reaction mixture consisted of 2X SYBR Green PCR Master Mix (Applied Biosystems), 900 nM of each primer, and 2 μL of cDNA. The conditions for PCR thermal cycling included an initial step at 95 °C for 5 min, 40 cycles at 95 °C for 20 s, 60 °C for 30 s, and 72 °C for 20 s. At the end of the reaction, a curve analysis was conducted. Using the Glyceraldehyde-3-phosphate dehydrogenase (GAPDH) gene that was amplified in each series of PCR experiments, the results were normalized. The relative expression of target mRNA was determined using the method of comparative Ct described by Livak and Schmittgen (2001) (Table 1).

**Table 1:** Primer sequences used for RT-PCR

Primer	Sequence	Accession No	Product length (bp)
ATF4	Forward: 5′- GCCATCTCCCAGAAAGT-′3 Reverse: 5′- AGGTGGGTCATAAGGTTTGG-′3	XM_039079942.1	365
IRE-1α	Forward:5′- TTGACTATGCAGCCTCACTTC -3′ Reverse: 5′- AGTTACCACCAGTCCATCGC -3′	XM_017597474.2	86
AMPK	Forward:5′-GGGATCCATCAGCAACTATCG-′3 Reverse: 5′- GGGAGGTCACGGATCAGG-′3	NM_019142.3	873
Splice xbp-1	Forward: 5′-CTGAGTCCGCAGCAGG-3′ Reverse: 5′-CTTGTCCAGAATGCCCAAAAGG-3′	NM_001271731.1	119
PERK	Forward: 5′- GTGAAGGTCGAGAGGCGTCG-′3 Reverse:5′—AATGCCGTATCCGATGTGGG-′3	NM_031599.2	249
PPAR-α	Forward: 5′- GCGGAGATCTCCAGTGATATC-′3 Reverse: 5′- TCAGCGACTGGGACTTTTCT-′3	XM_006237009.4	257
FAS	Forward:5′-TCGAGACACATCGTTTGAGC-3′ Reverse: 5′-CTCAAAAAGTGCATCCAGCA-3′	NM_017332.2	170
CHOP	Forward:5′-AGGAGAGAGAAACCGGTCCAA-3′ Reverse: 5′-GGACACTGTCTCAAAGGCGA-3′	XM_006241445.4	269
GAPDH	Forward: 5′- CTCCCATTCTTCCACCTTTG-′3 Reverse: 5′- CTTGCTCTCAGTATCCTTGC-′3	NM_017008.4	262

### Histopathological study

Liver tissue specimens from all animal groups which were carried out on five rats were obtained and preserved in 10% neutral buffered formalin. The specimens were then trimmed, washed, and dehydrated in ascending concentrations of alcohol, cleaned in xylene, embedded in paraffin, sectioned at 5–6-μm thickness, and stained with hematoxylin–eosin staining (H&E) according to Banchroft et al. (1996). The frequency and severity of lesions in the livers were evaluated semi-quantitatively, as reported earlier by Plaa (1982), using a scale where grade 0: no apparent injury, grade I: hepatocyte swelling, grade II: hepatocyte ballooning, grade III: lipid small bubbles in hepatocytes, and grade IV: hepatocellular apoptosis and necrotizing.

### Statistical analysis

The data was analyzed and significance tests were conducted using the statistical software SPSS (Statistical Program for Social Science) version 20.0, which includes a one-way ANOVA test followed by a post hoc test for multiple comparisons. All data is given as a mean of six values, with SE and the difference between means deemed significant if the difference is < 0.05.

## Results

### Effect of SFN on the body weight and liver injury of rats fed on HFD and/or exposed to IR

**Fig. 1:**
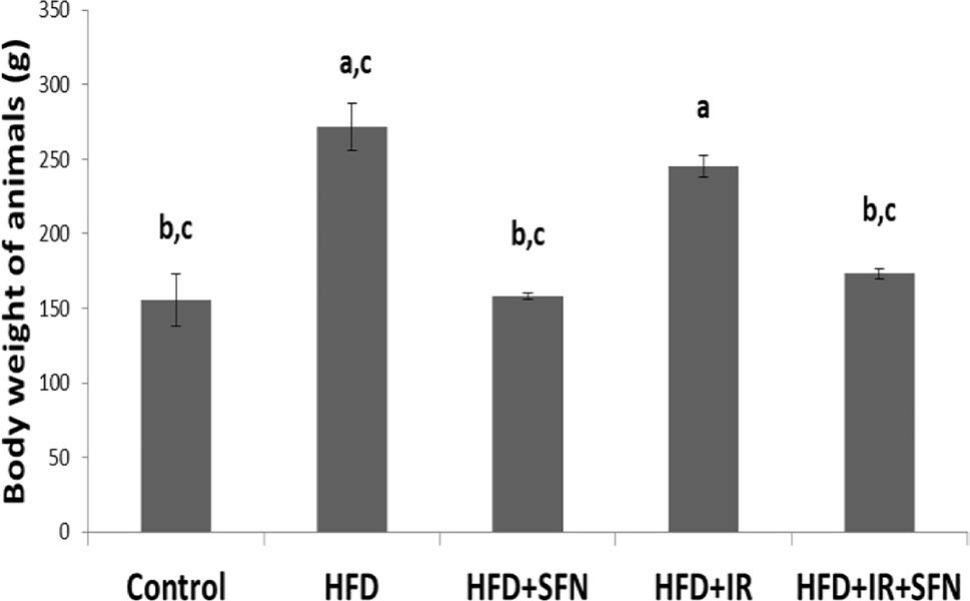
Effect of SFN on body weight of animals in rats fed with an HFD and/or exposed to IR. Each bar represents mean ± SEM (*n* = 6). ^a^Significant difference versus control group, ^b^Significant difference versus HFD group, ^c^Significant difference versus HFD + IR group. (*p* < 0.05, ANOVA)

The body weight of rats in each of the five experimental groups was monitored to assess the effects of SFN on obesity. During the experiment, the body weight of rats in the HFD and HFD + IR groups increased compared with that in the control group. SFN treatment reduced this body weight gain in the treated groups (HFD + SFN and HFD + IR + SFN) compared with that in the HFD and HFD + IR groups respectively (Fig. 1). Similarly, liver enzymes (ALT, AST, and ALP) are enzymes released from hepatocytes to the blood upon liver damage; these enzymes are considered the most sensitive indices reflecting liver cell damage. In contrast to the control group, the HFD group had a significant increase, but HFD + IR had higher levels in the serum activities of ALT, AST, and ALP. However, SFN supplementation ameliorated this damage as indicated by a significant decrease in the ALT, AST, and ALP activities in the intervention groups (HFD + SFN and HFD + IR + SFN) compared with those in the HFD and HFD + IR model groups respectively (Table 2).

**Table 2:** Effect of SFN on serum activities of ALT, AST, and ALP of rats fed on HFD and/or exposed to IR

Parameters	Groups				
	Control	HFD	HFD + SFN	HFD + IR	HFD + IR + SFN
ALT (U/ml)	32.5 ± 0.51^bc^	40.4 ± 2.15^abc^	31.5 ± 0.96^bc^	96.8 ± 1.02^ab^	43.2^c^ ± 0.36^ac^
AST (U/ml)	142.4 ± 0.7^bc^	161.3 ± 10.3^ac^	147.2 ± 0.6^c^	189.7 ± 0.8^ab^	150.9 ± 0.9^c^
ALP (U/ml)	22.4 ± 0.5^bc^	32.2 ± 0.4^a^	26.6 ± 1.6^abc^	32.5 ± 1.6^a^	26.0^c^ ± 0.4^abc^

Value is the mean ± *SEM* (*n* = 6). ^a^Significant difference versus control group, ^b^Significant difference versus HFD group, ^c^Significant difference versus HFD + IR group (*p* < 0.05, ANOVA)

### Effect of SFN on the metabolic parameters of rats fed on HFD and/or exposed to IR

To determine the effect of SFN on lipid metabolism, the levels of TG, TC, LDL-c, and FFA in the serum of the studied groups were measured. When rats subjected to HFD and/or IR were compared to those fed a normal diet, serum levels of TG, TC, LDL-c, and FFA increased significantly, while HDL-c levels decreased significantly. However, SFN supplementation to rats fed on HFD and/or exposed to IR displayed significant decreases in TG, TC, LDL-c, and FFA along with elevations in HDL-c levels compared to the animals in the HFD and HFD + IR groups. Meanwhile, to investigate whether treatment with SFN affects glucose metabolism, serum was collected to determine the levels of fasting blood glucose and fasting insulin. The HOMA-IR index was calculated based on the levels of fasting blood glucose and fasting insulin. A significant increase in the serum levels of fasting blood glucose and fasting insulin, as well as in the HOMA-IR index, was observed in the rats in the HFD and HFD + IR groups compared to the rats in the normal control group. In contrast, the levels of glucose and insulin, as well as the HOMA-IR index, were significantly reduced following SFN administrations in the HFD and HFD + IR + SFN groups (Table 3). This suggested that SFN could regulate lipid and glucose metabolism.

**Table 3:** Effects of SFN on lipid profile, glucose, insulin, and FFA of rats fed on a high-fat diet and/or exposed to IR

Parameters	Groups				
	Control	HFD	HFD + SFN	HFD + IR	HFD + IR + SFN
TC (mg/dl)	102.8 ± 1.72^bc^	169.0 ± 13.20^ac^	109.8 ± 4.90^bc^	220.1 ± 3.04^ab^	136.5 ± 4.02^abc^
TG (mg/dl)	87.3 ± 6.32^bc^	156.0 ± 8.50^ac^	81.7 ± 6.71^bc^	180.2 ± 8.90^ab^	103.1 ± 8.32^abc^
LDL-c (mg/dl)	27.3 ± 0.52^bc^	49.0 ± 4.3^ac^	29.0 ± 0.69^bc^	78.5 ± 3.65^ab^	39.72 ± 1.31^abc^
HDL-c (mg/dl)	16.7 ± 0.75^bc^	11.3 ± 0.80^ac^	18.8 ± 2.35^abc^	6.0 ± 0.15^ab^	11.9 ± 1.52^abc^
FFAs (mg/dl)	41.12 ± 1.7^bc^	152.0 ± 3.7^ac^	42.07 ± 0.3^bc^	203.1 ± 5.2^ab^	58.1 ± 1.3^abc^
Glucose mg/dl	94.22 ± 1.67^bc^	163.18 ± 2.73^ac^	93.40 ± 0.93^bc^	105.84 ± 0.95^ab^	96.90 ± 0.29^bc^
Insulin mIU/l	36.06 ± 1.17^bc^	43.73 ± 1.61^ac^	34.61 ± 0.95^bc^	39.95 ± 1.16^ab^	36.45 ± 1.22^b^
HOMA-IR	8.36 ± 0.03^bc^	18.12 ± .57^ac^	8.18 ± 0.29^bc^	10.22 ± 0.21^ab^	8.98 ± 0.26^bc^

Value is the mean ± *SEM* (*n* = 6). ^a^Significant difference versus control group, ^b^Significant difference versus HFD group, ^c^Significant difference versus HFD + IR group. (*p* < 0.05, ANOVA)

### Effect of SFN on hepatic oxidative stress of rats fed on HFD and/or exposed to IR

One of the most notable features of NAFLD is oxidative stress. Therefore, the effect of SFN on oxidative stress was assessed. Oxidative stress condition was assessed by determining the levels of MDA, SOD, CAT, and GSH in the liver tissue. In the present study, a significant increase in MDA level in the HFD rat group and a higher elevation in the HFD + IR group compared to the control group. On the contrary, significant decreases in the MDA concentration were observed in HFD + SFN and HFD + IR + SFN groups compared to the HFD and HFD + IR groups respectively. Meanwhile, SOD, CAT, and GSH are naturally produced cellular antioxidants which are accountable for decreasing oxidative stress. The activities of SOD and CAT and GSH content were significantly reduced in HFD and HFD + IR groups, in comparison with the control group. The intake of SFN to HFD and HFD + IR groups significantly restored all these antioxidant enzyme activities compared to the HFD and HFD + IR groups respectively (Table 4).

**Table 4:** Effects of SFN on oxidative stress biomarkers of rats fed on a high-fat diet and exposed to IR

Parameters	Groups				
	Control	HFD	HFD + SFN	HFD + IR	HFD + IR + SFN
MDA (µmol/gm tissue	147.9 ± 7.51^bc^	263.1 ± 4.4^ac^	125.2 ± 4.1^abc^	358.4 ± 13.8^ab^	160.2 ± 5.1^abc^
SOD (U/mg protein)	2.9 ± 0.15^bc^	1.3 ± 0.11^ac^	3.2 ± 0.17^bc^	0.8 ± 0.02^ab^	1.7 ± 0.11^abc^
CAT (U/mg protein)	2.1 ± 0.13^bc^	1.5 ± 0.32^a^	2.3 ± 0.12^bc^	1.4 ± 0.07^ab^	1.8 ± 0.09^bc^
GSH (µmol/g tissue)	59.8 ± 6.1^bc^	41.6 ± 2.17^ac^	73.4 ± 1.7^ac^	30.4 ± 1.8^ab^	51.7 ± 1.9^bc^

Value is the mean ± *SEM* (*n* = 6). ^a^Significant difference versus control group, ^b^Significant difference versus HFD group, ^c^Significant difference versus HFD + IR group. (*p* < 0.05, ANOVA)

### SFN declines the pro-inflammatory cytokine and adipokines of rats fed on HFD and/or exposed to IR

Another important stage of NAFLD is the inflammatory response. Therefore, the effect of SFN on HFD and/or exposure to IR-induced inflammatory response was measured. Pro-inflammatory adipokines (leptin and resistin) and pro-inflammatory cytokines (IL-1 β) were considered as potential metabolic syndrome serum markers. Hence, their levels were analyzed in the serum of control and experimental rats. The obtained data showed a significant increase in leptin, resistin, and IL-1β concentrations in the HFD, and highly elevated in the HFD + IR groups. However, treatment with SFN restored the altered levels of adipokines in rats fed on HFD and/or exposed to IR (Table 5**).**

**Table 5:** Effect of SFN on serum leptin, resistin, and IL-1β levels of rats fed on a high-fat diet and exposed to IR

Parameters	Groups				
	Control	HFD	HFD + SFN	HFD + IR	HFD + IR + SFN
Leptin (Pg/ml)	17.2 ± 0.7^bc^	103.7 ± 6.6^ac^	16.6 ± 0.35^bc^	149.9 ± 11.6^abc^	26.1 ± 0.22^bc^
Resistin (ng/ml)	4.92 ± 0.37^bc^	14.6 ± 0.3^ac^	4.17 ± 0.37^bc^	30.2 ± 0.02^abc^	6.74 ± 1.25^bc^
IL-1β (ng/ml)	37.2 ± 0.7^bc^	78.5 ± 5.1^ac^	30.5 ± 0.73^bc^	130.8 ± 4.5^abc^	54.4 ± 2.52a^bc^

Value is the mean ± *SEM* (*n* = 6). ^a^Significant difference versus control group, ^b^Significant difference versus HFD group, ^c^Significant difference versus HFD + IR group. (*p* < 0.05, ANOVA)

### Effects of SFN on the expression of adipogenesis regulators in the liver of rats fed on HFD and exposed to IR

**Fig. 2:**
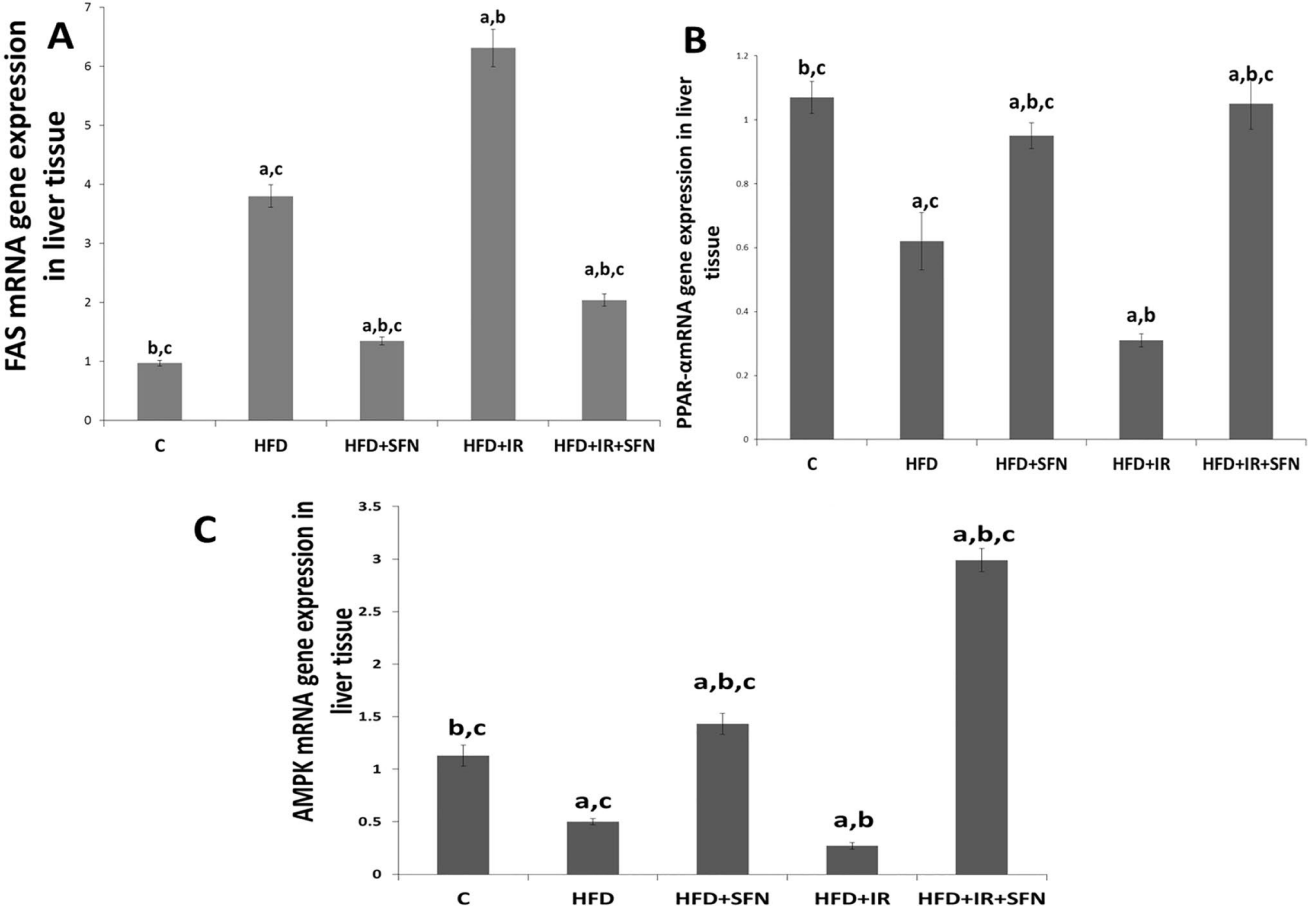
Effect of SFN on hepatic gene expression of (**A**) FAS, (**B**) PPARα, and (**C**) AMPK in HDF and HFD + IR groups. Each bar represents mean ± SEM (*n* = 6). ^a^Significant difference versus control group, ^b^Significant difference versus HFD group, ^c^Significant difference versus HFD + IR group. (*p* < 0.05, ANOVA)

To further understand how SFN improves fatty liver disease, we evaluated the expression of key genes encoding proteins that function in lipogenesis (FAS) fatty acid oxidation and (PPAR-α and AMPAK). The qRT-PCR results revealed that FAS gene expression increased significantly accompanied by a marked decrease in the mRNA expressions of PPARα and AMPAK in rats fed with HFD and/or exposed to IR compared to normal control. Interestingly, the administration of SFN to HFD and HFD + IR groups significantly regulated the expression of PPAR-α, AMPK, and FAS compared to HFD or HFD + IR groups respectively (Fig. 2).

### SFN regulated the expression of PI3K and Akt of rats fed on HFD and exposed to IR

**Fig. 3:**
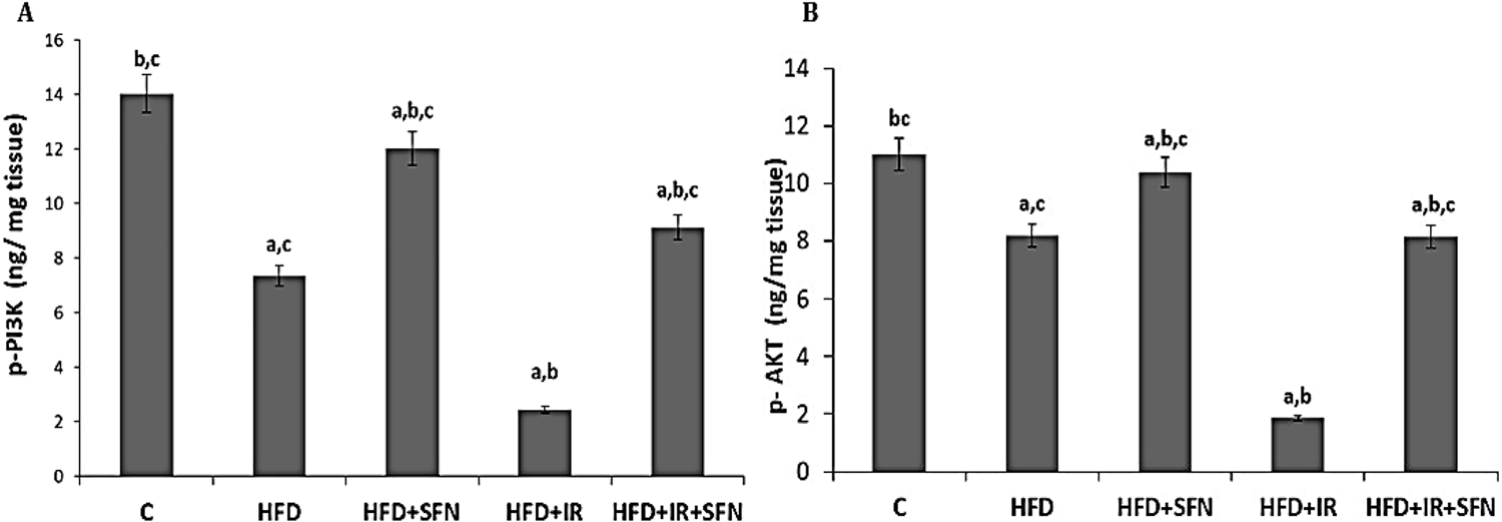
Effect of SFN on hepatic PI3K/AKT signaling. (**A**) P-AKT and (**B**) P-PI3K levels in HFD and HFD + IR groups. Each bar represents mean ± SEM (*n* = 6). ^a^Significant difference versus control group, ^b^Significant difference versus HFD group, ^c^Significant difference versus HFD + IR group. (*p* < 0.05, ANOVA)

Accumulating evidence indicates that dysregulation of the PI3K/AKT pathway in hepatocytes is a common molecular event associated with metabolic dysfunctions including the NAFLD and the pathogenesis of insulin resistance. The results of PI3K and Akt protein in liver tissue showed a significant decrease in the PI3K/AKT protein concentrations in the HFD and more reduction in the HFD + IR groups when compared to the control group. Additionally, the results showed a significant increase in these protein concentrations in the HFD + SFN and HFD + IR + SFN groups in comparison with the HFD and HFD + IR groups respectively. These results indicated that SFN could exert its protective effect against HFD and/or IR-induced hepatic damage by downregulation of the PI3K/Akt signaling pathway (Fig. 3).

### Effect of SFN on the expression of hepatic ER Stress biomarkers and CHOP of rats fed on HFD and/or exposed to IR

**Fig. 4:**
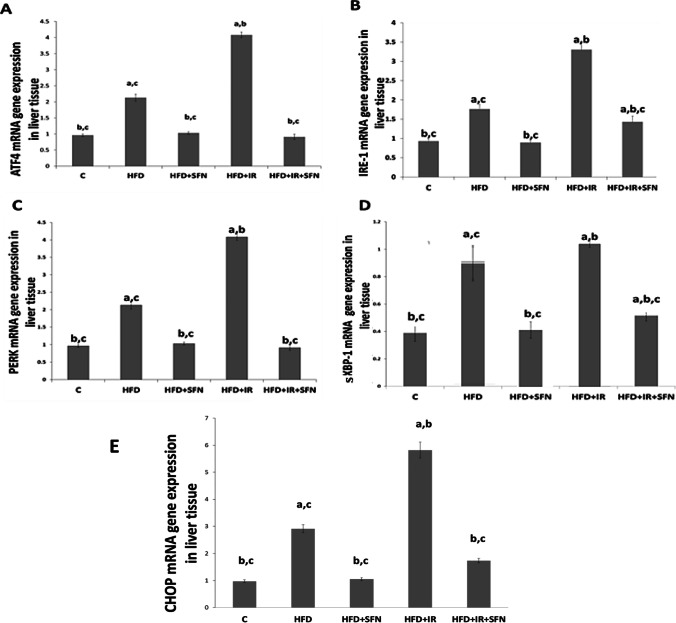
Effect of SFN on hepatic mRNA expression of gene-related ER stress. (**A**) ATF4, (**B**) IRE-1α, (**C**) PERK, (**D**) sXBP-1, and (E) CHOP. Each bar represents mean ± SEM (*n* = 6). ^a^Significant difference versus control group, ^b^Significant difference versus HFD group, ^c^Significant difference versus HFD + IR group (*p* < 0.05, ANOVA)

The ER stress markers (ATF4, IRE-1α, PERK, sXBP-1, and CHOP) are crucial proteins involved in the pathogenesis of inflammation and insulin resistance; hence, we investigated their gene expression in the liver of experimental rats. In the present study, rats fed on HFD and exposed to IR exhibited high gene expression of IRE1, PERK, ATF4, and sXBP-1 compared to control, whereas the expression of tissue CHOP was significantly downregulated. On the other hand, the SFN supplementation to rats fed on HFD and/or exposed to IR significantly regulated the expressions of ATF4, IRE-1α, PERK, sXBP-1, and CHOP compared to HFD and HFD + IR groups (Fig. 4).

### Histology finding

**Fig. 5:**
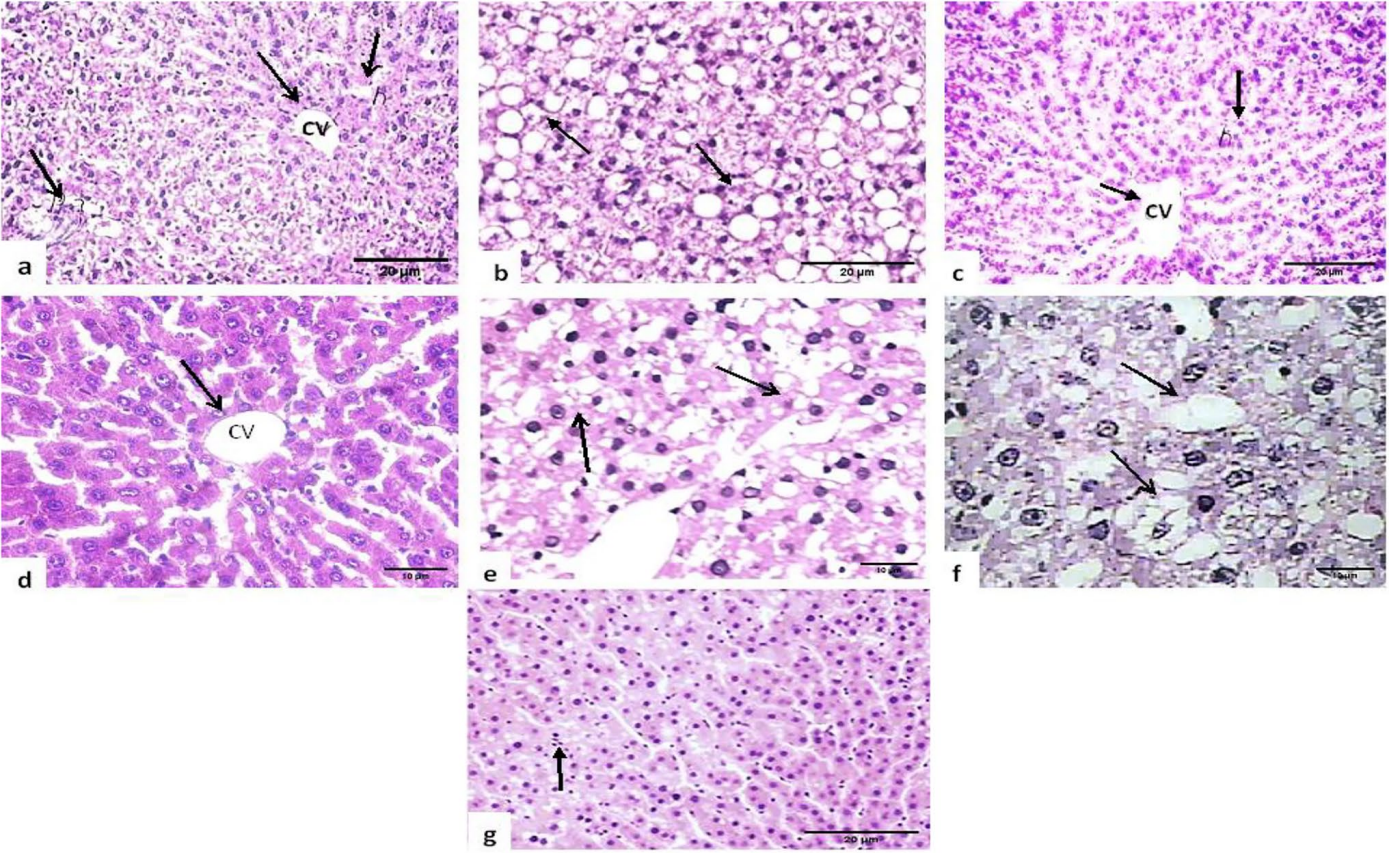
A photomicrograph of hepatic rat tissue sections (*n* = 6 from each group) stained with H&E (200 × and 400 ×) shows the control group image (**a**, **d**) normal organization of hepatic lobule structure of the central vein (CV) and portal area (Pa) with the surrounding hepatocytes (**h**) arrow (grade 0). HFD group image (**b**) showed fatty degeneration, severe macrovesicular steatosis with a few foci of inflammatory cells (grade III), and HFD + SFN group image (**c**) showed the normal appearance of the hepatic cord radiated from the central vein (arrow) (grade 0), HFD + IR group image (**e**, **f**) shown the appearance of many vacuolated areas (arrow), hydropic and fatty degeneration (grade IV) (NAFLD liver), and HFD + IR + SFN group image (**g**) treatment markedly attenuated the histopathological characteristics of NAFLD observed in the model group (grade I)

Photomicrographs of normal liver samples are shown in Fig. 5a–f. Photomicrographs of the liver from rats fed on a high-fat diet showed the presence of many vacuolated areas, many fat cells, and dilatation of the central vein. The liver of an HFD rat subjected to gamma-rays (NAFLD model) revealed an increase in the appearance of many vacuolated areas, as well as hydropic and fatty degeneration. The liver of HFD rats treated with SFN showed a reduction in fat cells and less dilatation of the central vein. In comparison to the control group, NAFLD rats treated with SFN showed some normalization, with the fewest intracellular micro-vesicular steatosis, undamaged architecture, and no inflammatory foci as described in Table 6.

**Table 6:** The grading standards for liver injury

Groups	Liver injury grade	Description of pathology
Control	0	Indicates minimal or no evidence of injury
HFD	III	Lipid droplets in hepatocytes
HFD + SFN	0	Indicates minimal or no evidence of injury
HFD + IR	IV	Apoptosis and necrosis of hepatocytes
HFD + IR + SFN	I	Hydropic degeneration

## Discussion

The results originating from this investigation confirm the ability of SFN to alleviate hepatic steatosis and liver damage in rats chronically fed HFD, which supports some previous studies conducted in the NAFLD model (Wu et al. 2021; Li et al. 2021). However, the molecular mechanisms underlying the beneficial effect of SFN in the treatment of NAFLD remain controversial. The novelty of our data is that they are the first to show that this protection is mediated, at least by activating AMPK, which subsequently ameliorated hepatic steatosis and oxido-inflammatory damage by regulating ER stress, and lipid metabolism, and glucose homeostasis.

In the present study, SFN administration significantly reduced the body weight gain in the HFD and HFD + IR groups. To evaluate whether the hepatic steatosis was induced in this model, we first investigated the liver enzymes (ALT, AST, and ALP). Liver enzymes are usually used as a sign of liver impairment and as surrogate diagnostic markers for NAFLD, apart from liver biopsy as a gold standard for NAFLD diagnosis. Any damage to the hepatocytes increases the activity of these enzymes in the liver before being transported into the bloodstream, and thus increases the levels of the enzyme in the serum (Eliades et al. 2013). The data showed that feeding rats with HFD and/or HFD + IR groups caused remarkable increases in the liver enzymes compared to those in the control group; on the other hand, SFN administration significantly rescued the markedly increased serum levels of ALT, AST, and ALP in HFD and HFD + IR groups.

Nonalcoholic fatty liver (NAFL) is mainly characterized by fat deposition in hepatocytes, visible under light microscopy as small droplets inside the cytoplasm. Thus, therapy based on reducing lipid accumulation is ideal for treating NAFLD (Wang and Malhi 2018). The previous study suggests that insulin resistance status is highly related to the alteration of lipid mechanisms, accompanied by reduced serum HDL as well as increased LDL and TG levels. In the present study, a significant increase was observed in serum TG, TC, LDL-c, and FFA, accompanied by a significant decrease in HDL-c compared to both groups of HFD and HFD + IR groups. Interestingly, SFN administration in the HFD + IR group significantly reversed all these undesirable changes, which is in agreement with previous findings (Li et al. 2021). Moreover, the reduction in the lipid profile in the serum of rats treated with SFN (HFD + SFN and HFD + IR) was associated with downregulation in the gene expression of FAS, confirming that SFN could reduce the fatty acid chain synthesis by suppressing FAS expression. The anti-hyperlipidemic activity of SFN was supported by the study of Lei et al. (2019), who suggested that the anti-hyperlipidemic property of SFN might be attributed to its lipolysis activity by transcriptionally upregulating adipose triglyceride lipase and hormone-sensitive lipase in HHL-5 cells.

The exact pathogenesis of NAFLD is still unknown, but accumulating evidence has indicated important roles for oxidative stress, insulin resistance, endoplasmic reticulum stress, and chronic inflammation, and these factors always interact with each other and finally lead to the occurrence and development of NAFLD. Oxidative stress is often initiated by abundant production of ROS and is considered an important contributor to hepatocyte injury associated with NAFLD (Li et al. 2018). This is in line with our findings in which significant increases in the oxidative stress markers such as MDA were accompanied by decreased activities of catalase, SOD, and GSH content in the HFD and HFD + IR groups compared to the control group, suggesting the presence of oxidative stress that may play an essential role in the insulin resistance in the HFD and HFD + IR groups. In this context, the significant increase in liver MDA could be due to the interaction of ionizing radiation with water, which produces a variety of reactive oxygen species (ROS), including hydroxyl radical (•OH), hydrogen peroxide (H2O2), superoxide radical (•O_2_), and subsequently oxygen (O_2_) as an attack on the fatty acid composition of membrane lipids (Hassan et al. 2021). Nevertheless, SFN treatment reduced the oxidative stress in rats fed on HFD and/or exposed to an IR, as detected by the activation of key enzymes involved in the balance of the redox state, such as catalase, SOD, and GSH. Considering the role played by SOD and catalase in the protection of cells against oxidative damage, the increased activity of these enzymes following SFN treatment suggests a decreased hepatic oxidative stress and insulin resistance in SFN-treated rats (Wang and Chan 2006).

Meanwhile, insulin resistance is one of the variables in the metabolic syndrome associated with NAFLD and it is defined as a decrease or insufficient insulin sensitivity in the target tissues, such as muscle, adipose tissue, and liver, towards glucose uptake from the blood (Petersen and Shulman 2018). Previous data showed that HFD-fed animals demonstrated a reduction in insulin sensitivity (Kuipers et al. 2019; Meijer and Barrett 2021). This is attributed to the excessive free fatty acids derived from HFD, which inhibit insulin binding, degradation, and function and hence cause a decrease in glucose uptake from the blood (Ter Horst et al. 2017). The HOMA-IR index is used to assess systemic insulin resistance, and higher HOMA-IR values indicate a higher degree of insulin resistance. Our findings showed that rats in the HFD and HFD + IR groups demonstrated insulin resistance as indicated by significantly elevated values of HOMA-IR relative to the control group, which was confirmed by the previously reported studies. However, the elevated HOMA-IR index values that were associated with HFD administration were restored to normal control levels after SFN treatment.

Current research believes that insulin resistance plays an important role in the pathogenesis of NAFLD. The PI3K/Akt signaling pathway is one of the main downstream pathways of insulin, and Akt is the key signaling transduction molecule in the PI3K pathway. Physically, insulin induces the upstream activation and then Akt phosphorylation, which further mediates glycogen synthesis, glycolysis, glucose transporter, protein synthesis, and lipid synthesis. Also, some research demonstrated that Akt could directly inhibit the gene expression of fatty acid oxidation and thus regulate liver lipid metabolism. Although much evidence indicates that activation of the PI3K/AKT pathway is associated with marked accumulation of intracellular lipid droplets and promotion of NASH to fibrosis, some studies have revealed that PI3K/Akt activation is beneficial for ameliorating insulin resistance, oxidative stress, and lipid accumulation (Li et al. 2018). In the current study, HFD and/or ionizing radiation exposure inhibited the activity of PI3K/Akt proteins, which in turn induced significant insulin resistance, as detected by the significant increase in the HOMA-IR index. Additionally, studies also demonstrated that some natural products ameliorated NAFLD by regulating the PI3K/Akt pathway (Matsuda et al. 2013; Li et al. 2018), which was in accordance with our results that SFN could reduce insulin resistance by activating the PI3K/Akt phosphorylation.

Furthermore, previous studies have presented various concrete data on the role of adipose tissue as a key endocrine organ that mediates the metabolic activities of the brain, muscle, and cardiovascular system (Antuna-Puente et al. 2008; Kamada et al. 2008). The adipocytokines such as IL-1β, leptin, and resistin released by the adipocytes control insulin sensitivity and inflammation, which also take part in the pathogenesis of NAFLD and its progression to NASH (Fjære et al. 2019; Silva et al. 2020). In the present study, we showed that HFD feeding and IR exposure significantly increased the levels of IL-1β, leptin, and resistin compared to the control group. In contrast, SFN administration significantly reduced this parameter as well as increased insulin sensitivity, which is in accordance with previous studies reported in animal and clinical trials (Kujawska-Luczak et al. 2018; Suleiman et al. 2020).

Dysfunction of the ER, the main cellular compartment involved in secretory and transmembrane protein folding, calcium homeostasis, and lipid biogenesis, is involved in metabolically driven NAFLD pathologies through the activation of ER stress signaling, during which the expression of FAS is upregulated (Ashraf and Sheikh 2015; Huang et al. 2010). In this study, the expression of IRE-1α, sXBP1, PERK, ATF4, and CHOP in response to ER stress was markedly increased in HFD and HFD + IR, but SFN treatment significantly reversed the increased activation of this gene expression. Taken together, the results reveal that SFN treatment regulates lipid metabolism by suppressing the ER stress in NAFLD. We investigated the mechanism underlying the attenuation of lipid metabolism disorders by SFN through inhibition of ER stress. AMPK, as an energy sensor, contributes to keeping cellular energy homeostasis (Hardie 2008). Activated AMPK abolishes the lipid synthesis process and reduces TG production in the liver (Yang et al. 2014). Previous research indicates that activation of AMPK inhibits ER stress (Kim et al. 2015) and lipogenesis and stimulates fatty acid oxidation by inhibiting the expression of lipid metabolism-related proteins (Zhou et al. 2017). FAS has been identified as an important target of AMPK. Phosphorylation of AMPK may downregulate the expression of FAS, ultimately leading to the inhibition of lipid synthesis (LiY et al. 2011). Moreover, AMPK has been reported to positively regulate FA oxidation by activating PPARα (Lee et al. 2006; Choi et al. 2014). Additionally, there is evidence to indicate that AMPK activates the PI3K/Akt pathway by inhibiting the phosphorylation of insulin receptor substrate-1 (Zheng et al. 2015).

Consistent with a previous study by Docrat et al. (2018), the livers of HFD and HFD + IR rats in the present study exhibited a significant downregulation in AMPK gene expression compared with the control group. The PI3K/Akt levels, as well as PPARα gene expression levels, were also reduced in the model groups compared with the control. However, SFN treatment for 4 weeks significantly reversed the decreased levels of AMPK, PI3K/Akt, and PPAR-α, indicating that SFN can regulate the expression of AMPK and its downstream targets.

Overall, the results of the present study suggest that SFN could effectively prevent the progression of NAFLD in a rat model, as evidenced by its ability to attenuate the HFD and/or ionizing radiation-induced increases in serum levels of liver enzymes, lipids, glucose homeostasis, and inflammatory adipokines. Mechanistically, the results also show that SFN regulated lipid metabolism, insulin resistance, and ER stress in the liver via the AMPK-dependent upregulation of PPAR-α, PI3K/AKT, and their target proteins. In conclusion, the key finding from the present study was that SFN reduced body weight and covalently inhibited the chaperones’ activity and could disconnect the transduction of ER stress signals from an inflammatory response and lipid metabolism. It can be concluded that sulforaphane could work as an antioxidant and anti-inflammatory agent, stressing the need for an SFN agent in the management of obesity and irradiated patients to protect or at least mitigate the therapy’s side effects.
